# Kenya Trypanosomiasis Research Institute Cryobank for Human and Animal Trypanosome Isolates to Support Research: Opportunities and Challenges

**DOI:** 10.1371/journal.pntd.0002747

**Published:** 2014-05-22

**Authors:** Grace A. Murilla, Kariuki Ndung'u, John K. Thuita, Purity K. Gitonga, Daniel T. Kahiga, Joanna E. Auma, Johnson O. Ouma, Jane J. Rutto, Joseph M. Ndung'u

**Affiliations:** 1 Kenya Agricultural Research Institute – Trypanosomiasis Research Centre (KARI-TRC), Kikuyu, Kenya; 2 Foundation for Innovative New Diagnostics (FIND), Geneva, Switzerland; Yale School of Public Health, United States of America

## Introduction

Human African trypanosomiasis (HAT) is classified in the category of the most neglected tropical diseases. In man, the disease is caused by two tsetse (*Glossina* spp.)-transmitted trypanosome subspecies: *Trypanosoma brucei gambiense*, which is responsible for the chronic form of HAT in West and Central Africa, and *T. b. rhodesiense*, which causes acute disease in eastern and southern Africa. African animal trypanosomiasis (AAT) is caused by various trypanosome species, the major ones being *T. vivax, T. congolense*, and *T. evansi* [1]. Current diagnostic tools are inadequate and diagnosis is complicated, whereas the drugs for treatment are highly toxic and not very effective; patients die if untreated [2]. In 2005, an annual prevalence of 50,000–70,000 cases per year and incidence rates of 15,000–17,000 cases per year were reported [3]. Although recent data from the World Health Organization (WHO) shows that the number of reported cases of HAT declined to less than 10,000 in 2009, leading to speculation that the disease could be eliminated [4], [5], there is great need to maintain vigilance.

The East African Trypanosomiasis Research Organization (EATRO) was established to carry out research and develop technologies for effective control of trypanosomiasis. In view of this, a trypanosome cryobank was established in Tororo, Uganda, in the mid-1950s to provide materials for research. At that time, dry ice was used as a refrigerant, but in 1977 it was replaced with liquid nitrogen. Following the collapse of the East African Community in 1977, the Kenya Trypanosomiasis Research Institute (KETRI) was established to take over the functions of tsetse and trypanosomiasis research in Kenya. The cryobank was therefore transferred to KETRI during this period. In 2003, following a reorganization of research institutions by the Government of Kenya, KETRI was merged with the Kenya Agricultural Research Institute (KARI) and renamed the Trypanosomiasis Research Centre (KARI-TRC). KARI-TRC continued with all the research programmes and activities that were being carried out under KETRI, including collection and preservation of trypanosome stabilates. The institution developed a policy on stabilate collection by scientists and clinicians for cryopreservation. We describe the establishment of the cryobank and procedures used in cryopreservation of stabilates and summarize the data (numbers and types) on trypanosome species stored in the cryobank, which are available for use in research by the scientific community.

**Figure 1:**
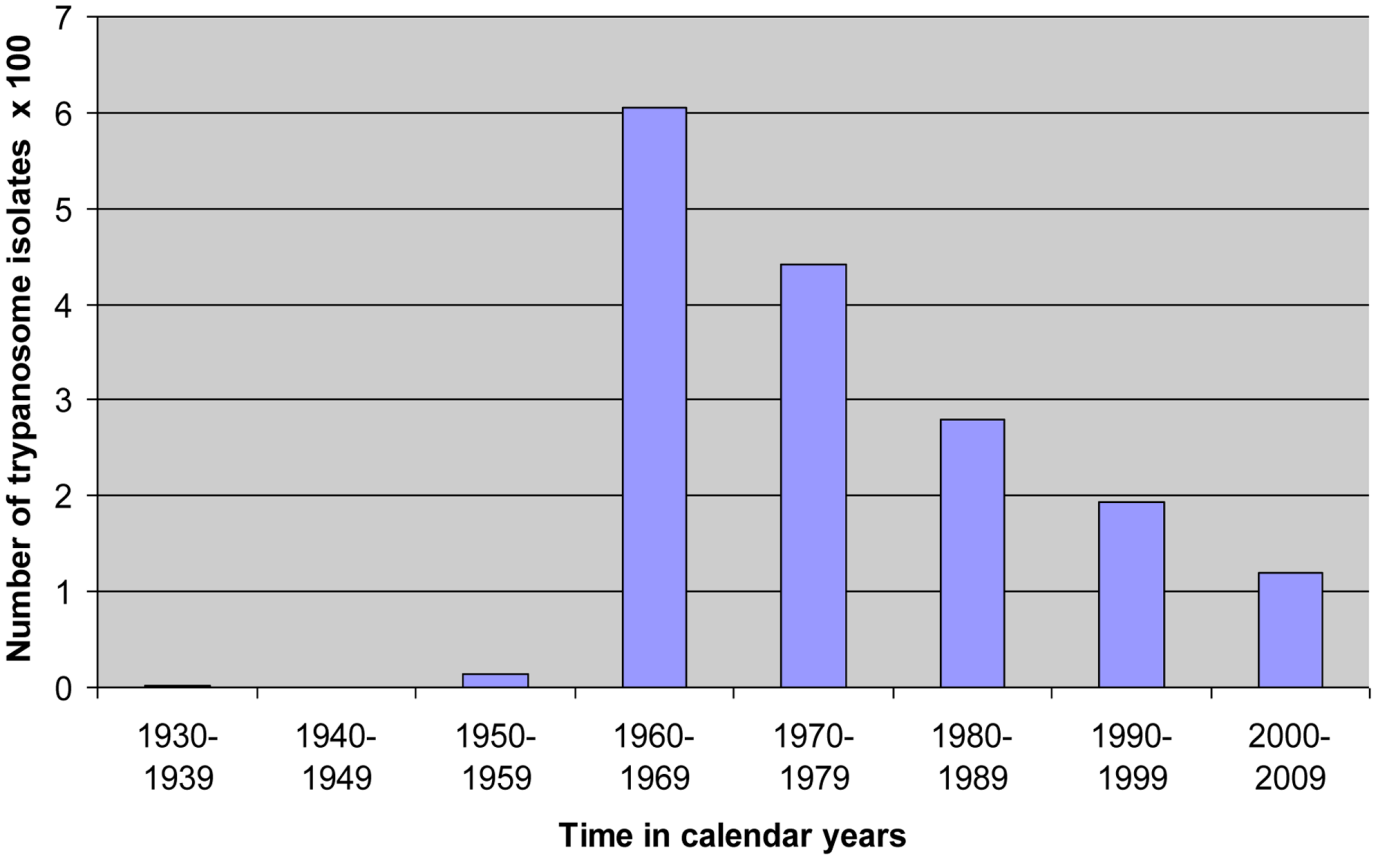
Number of primary trypanosome stabilates collected, preserved, and stored at the Kenya Trypanosomiasis Research Institute cryobank.

The cryobank contains 2,347 stabilates, including 1,747 primary isolates, out of which there are 42 mixed infections and one miscellaneous *Herpetomonas muscorum*, and 600 derivatives, including six mixed infections. Primary isolates were collected mainly from countries in the eastern Africa region, including Kenya, Uganda, Tanzania, Sudan, and Ethiopia. However, collections or donations from countries outside the region, including Nigeria, Mozambique, Botswana, Germany, and South America, have been added as part of collaborations between KARI-TRC and other institutions around the world. The stabilates were isolated between 1934 and 2010. The majority of the stabilates were recovered between 1960 and 1970 (Figure 1), the same period when some of the worst epidemics occurred, after which the numbers added have been on the decline. The period from 1940 to 1949 coincided with World War II, when the work on trypanosomiasis research and control stalled: the laboratories in eastern Africa that were the source of isolates were closed, only to resume after 1945 when the war came to an end.

### Trypanosomes

Trypanosomes are extracellular protozoan parasites which cause disease in humans and animals.

Isolation and cryopreservation of new trypanosome strains from patients in different HAT foci ensures availability of these stabilates for use in parasitological, biochemical, molecular, serological, and pharmacological studies many years after their isolation from the host. Brun et al. [1] observed that one of the major obstacles in the elucidation of the factors responsible for relapses after melarsopol treatment was lack of recent *T. b. gambiense* isolates from patients from various endemic areas where the problem had been reported. The WHO steering committee on human African trypanosomiasis treatment and the East African Network for Tsetse and Trypanosomiasis (EANETT) have therefore recommended that collection of stabilates be a continuous activity in order to monitor the occurrence and spatial distribution of treatment failure [6]. Since its inception, KETRI has established an institutional policy of encouraging collection of stabilates by scientists and clinicians for cryopreservation. In this paper, we describe the establishment of the cryobank and summarize the data (numbers and types) on trypanosome species stored in the cryobank, which are available for research by the wider scientific community.

### Existing data

An electronic database has been developed for the existing data and can be accessed through the KARI website (www.kari.org), which is currently being updated, and the WIPO Re:Search website (www.wipo.int/research/en/partnership/). The data is categorized into human, animal, tsetse fly, derived, and isolates characterized by drug sensitivity and molecular techniques. The total number of stabilates, including localities and period of isolation, is shown in Table 1.

**Table 1:** Primary trypanosome isolates collected from various countries and stored at the Kenya Trypanosomiasis Research Institute cryobank.

		Species of Trypanosomes: Number and period of isolation											
Country	*Isolate/Year*	*Tbb*	*Tb subgroup*	*Tbr*	*Tbg*	*T. congolense*	*T. vivax*	*T. evansi*	*T. simiae*	*T. theileri*	*T. lewesi*	UC	Mixed
**Kenya**	**No**	101	194	274	-	107	166	89	3	-	-	-	29
	**Year**	1961–2001	1961–2006	1958–2009	-	1961–2008	1969–2009	1968–2003	1970	-	-	18	1970–2006
**Uganda**	**No**	1	238	123	22	82	64	-	-	2	8	14	5
	**Year**	1968	1960–1983	1959–2004	1959–2002	1955–1983	1961–1972	-	-	1972–1973	1966	14	1955–1971
**Tanzania**	**No**	-	57	7	-	35	-	-	-	-	-	-	9
	**Year**	-	1966–1974	1934, 1959–1974	-	1966–1974	-	-	-	-	-	-	1966–1974
**Botswana**	**No**	-	-	2	-	-	-	-	-	-	-	-	-
	**Year**	-	-	1960		-	-	-	-	-	-		-
**Sudan**	**No**	-	-	-	26	-	-	2	-	-	-	-	-
	**Year**	-	-	-	1982–2003	-	-	1973	-	-	-	-	-
**Mozambique**	**No**	-	-	2	-	-	-	-	-	-	-	-	-
	**Year**	-	-	1980, 1983	-	-	-	-	-	-	-	-	-
**Nigeria**	**No**	-	-	-	-	-	4	-	-	-	-	-	-
	**Year**	-	-	-	-	-	1970–1973	-	-	-	-	-	-
**Zambia**	**No**	-	-	-	-	2	-	-	-	-	-	-	-
	**Year**	-	-	-	-	1981	-	-	-	-	-	-	-
NDA	**No**	2	21	8	-	17	5	1	-	-	-	3	2
	**Year**	1961	-	-	-	1962–1985	1961	-	-	-	-	-	2
**Total**		**104**	**510**	**416**	**48**	**243**	**240**	**92**	**3**	**2**	**8**	**35**	**45**

**Key:** *Tbb  =  Trypanosoma brucei brucei; Tbr  =  Trypanosoma brucei rhodesiense; Tbg  =  Trypanosoma brucei gambiense*; UN  =  unclassified; NDA  =  no data available; *: academic institutes from Germany (B. Weitz Lister and Berlin University) donated these stabilates to the KETRI cryobank. Of the unclassified, 14 are from tsetse flies, two from humans, and two from unknown hosts.

### Human infective trypanosomes

Of the 1,745 primary stabilates in the cryobank, 416 (25%) are *T. b. rhodesiense*, of which 60 were isolated from cerebrospinal spinal fluid (CSF), and 48 (3%) are *T. b. gambiense* (Table 1). The *T. b. gambiense* isolates were collected mainly from Uganda and South Sudan. Four hundred and twenty–seven (92%) of the human infective parasites were recovered from patients, four from animals (Table 2), and 33 from tsetse flies (Table 3). The human infective trypanosomes include three which were isolated from one family consisting of a mother, son, and grandson in Lambwe Valley, Kenya.

**Table 2:** Animal hosts from which various trypanosomes were isolated and stored at the Kenya Trypanosomiasis Research Institute cryobank.

	*Tbb*	*Tb subgroup*	*Tbr*	*T. congolense*	*T. vivax*	*T. evansi*	*T. theileri*	*T. simiae*	*T. lewesi*	*UC*	*Mixed*	*Total*
Cattle	85	247	0	119	137	0	2		0	10	21	621
Goat	0	6	0	6	6	0	0	0	0	0	1	19
Sheep	1	8	1	24	3	0	0	0	0	0	7	44
Pig	1	5	0	0	0	0	0	0	0	0	0	6
Camel	0	3	0	1	2	92	0	0	0	0	0	98
Donkey	0	1	0	1	0	0	0	0	0	3	0	5
Cat	0	0	0	0	0	0	0	0	0	0	2	2
Dog	3	6	0	1	0	0	0	0	0	1	0	11
Wildlife	2	40	3	12	0	0	0	0	0	0	5	62
Lizard	0	0	0	0	0	0	0	0	0	3	0	3
Rat	0	0	0	0	0	0	0	0	8	0	0	8
HNI	1	7	4	2	2	0	0	0	0	2	0	18
**Total**	**93**	**323**	**8**	**166**	**150**	**92**	**2**	**0**	**8**	**19**	**36**	**897**

**Key:** Tbb  =  Trypanosoma brucei brucei; Tb  =  Trypanosoma brucei; Tbr  =  Trypanosoma brucei rhodesiense; UC  =  unclassified; HNI  =  host of isolation not indicated.

**Table 3:** Trypanosome stabilates isolated from tsetse flies and stored at the Kenya Trypanosomiasis Research Institute Cryobank.

	*Tbb*	*Tb subgroup*	*Tbr*	*T. congolense*	*T. vivax*	*T. simiae*	*UC*	*Mixed*	*Total*
***Gb***	0	0	0	8	8	3	1	4	24
***Gff***	0	24	0	1	14	0	0	0	39
***Gfu***	0	1	0	0	2	0	0	0	3
***Gmm***	0	8	31	1	1	0	0	0	41
***Gpp***	1	6	1	0	0	0	10	0	18
***Gp***	8	133	1	66	58	0	3	4	273
***GSWY***	0	8	0	0	7	0	0	0	15
**NDA**	2	5	0	1	0	0	0	1	9
**Total**	**11**	**185**	**33**	**77**	**90**	**3**	**14**	**9**	**422**

**Key: Gb**  =  G. brevipalpis; **Gff**  =  G. fuscipes fuscipes; **Gfu**  =  G. fuscipleuris; **Gmm**  =  G. morsitans morsitans; **Gpp**  =  G. palpalis palpalis; **Gp** = G. pallidipes; **GSWY**  =  G. swynertoni; NDA  =  no data available; UC  =  unclassified.

A small number of the stabilates have been characterized using PCR, procyclic transmission test, and isoenzyme techniques (Table 4) and assessed for drug sensitivity profiles (Table 5). The data shows that only 22% of the cryobank primary isolates have been characterized at the molecular level (Table 4), indicating that there are numerous opportunities for new studies utilizing the uncharacterized stabilates. The infectivity characterization of *T. b. gambiense* isolates collected from Sudan revealed five isolates which successfully infected Swiss white mice [7].

**Table 4:** Trypanosome stabilates characterized using PCR, procyclic transmission test, and isoenzyme techniques.

		*Technique used in the characterization*			
*Species of tryps*	*Number of stabilates*	PCR	Procyclic Transmission Test (PTT)	Isoenzyme	References
***T. evansi***	32 (35%)	+	+	+	[8]–[11]
***T. vivax***	9 (4%)	-	-	+	[12]
***T. b. gambiense***	17 (35%)	+	-	+	[7], [11], [13]
***T. b. rhodesiense***	25 (5%)	+	-	+	[14], [15]
***T. simiae***	1 (33%)	+	-	-	[16]
***T. brucei***	274 (45%)	+	+	-	[17], [18]

**Key:** +  =  test was performed; -  =  test was not performed; numbers in parentheses  =  total number of stabilates in the cryobank.

**Table 5:** Drug resistant trypanosome stabilates stored at the Kenya Trypanosomiasis Research Institute cryobank.

Species	*Stabilates*	*Trypanocidal Drug(s)*	*Dose level*	*References*
T. b. rhodesiense*	KETRI 2538, 2694, 2709, EATRO 237, 243, 240, & 1992	Melarsoprol	4×20 mg/kg	[1]
*T. b. rhodesiense*	EATRO 243, KETRI 2708, KETRI 2538	Melarsoprol	1, 5, & 10 mg/kg	[19]
*T. b. rhodesiense*	3150a, 3151a & 3152a	Melarsoprol	3.6 mg/kg×4	KARI-TRC, unpublished data
T. b. rhodesiense*	EATRO 243	Melarsoprol; Melarsen oxide	1.0, 2.0, 5.0, & 10 mg/kg; 1.0, 5.0 & 10 mg/kg	[20]
*T. b. rhodesiense*	KETRI 2002	Melarsoprol, Melarsen oxide	1.0 mg/kg; 1.0, 2.0, 5.0, & 10 mg/kg	[20]
*T. b. rhodesiense*	KETRI 2538	Melarsoprol, Melarsen oxide	1.0, 2.0, 5.0, & 10 mg/kg	[20]
T. b. rhodesiense*	EATRO 243, 1992, & KETRI 2538	Diminazene	10 mg/kg	[19]
*T. b. rhodesiense*	EATRO 243, 269, 1992, & KETRI 2538	Pentamidine	2 mg/kg	[19]
*T. b. rhodesiense*	EATRO 265, 269, & KETRI 2538	DFMO	2% and 4%	[19]
T. b. rhodesiense*	EATRO 237, KETRI 2538, 2694	Samorin, Diminazene, Homidium Mel B	1.0 mg/kg; 20 mg/kg; 1.0 mg/kg; 10 mg/kg	[21]
T. b. rhodesiense*	KETRI 3530	Diminazene; Homidium	20 mg/kg; 1.0 mg/kg	[21]
T. b. rhodesiense*	KETRI 2579, 2630, 2628, 2606, 2605, 2604, & 2653	Suramin*	Dose level administered to patients not indicated.	KARI-TRC, unpublished data
*T. congolense*	KETRI 2776	Diminazene	3.5 & 7.5 mg/kg	[22]
*T. congolense*	KETRI 2880	Diminazene, Samorin	7.0 mg/kg; 0.5–1.0 mg/kg	[22]
*T. congolense*	KETRI 2883	Diminazene	10.5 mg/kg	[23]
*T. evansi*	EATRO 1188, KETRI 2411, 2415, & 2424	Diminazene, Samorin, Ethidium, Novidium	3.5, 7.0, 10.5 mg/kg; 0.5, 1.0, 2.0, 4.0, & 8.0 mg/kg; 1.0, 2.0, 3.0 mg/kg; 1.0, 2.0, 3.0 mg/kg	[24]

Other than KETRI 2538, which is molecularly characterized (not published), molecular characterization of the other isolates is not available. **Key**: *  =  these isolates were recovered from cases of treatment failure following suramin chemotherapy; a  =  stabilates which were made resistant to melarsoprol in the laboratory; EATRO  =  East African Trypanosomiasis Research Organization; KETRI  =  Kenya Trypanosomiasis Research Institute; DFMO  =  difluoromethylornithine.

Also available are trypanosome stabilates isolated from various body fluids, including blood, CSF, peritoneal fluid, and lymph nodes.

### Isolates from animals

The cryobank contains 897 trypanosome stabilates that were recovered from different species of animals, including cattle, sheep, goats, camels, pigs, wildlife, rats, and lizards (Table 2). The stabilates consist mainly of *T. brucei* subsp., *T. congolense*, and *T. vivax*. Cattle were the main source of all animal-derived stabilates (71%), whereas camels and wildlife contributed 11.5% and 7%, respectively (Table 2). The wildlife-derived stabilates were isolated from lions, wildebeest, zebra, bushbuck, grey ducker, and impala, among others, before 1974. A total of 18 and 37 stabilates were isolated from goats and sheep, respectively, while a number of miscellaneous species, including *T. theileri* (2) and *T. lewisi* (8), were isolated in Uganda between 1966 and 1973. There are 45 stabilates (Table 1) of mixed infections, mainly of *T. congolense* with *T. vivax*, *T. brucei*, or *T. simiae*. The list also includes species of trypanosomes whose host of isolation was not documented.

### Isolates from tsetse flies

The cryobank contains 422 primary trypanosome isolates that were collected from different species of tsetse flies including *G. pallidipes*, *G. brevipalpis*, *G. morsitans morsitans*, *G. palpalis palpalis*, *G. fuscipes fuscipes*, *G. fuscipleuris*, *G. swynertoni*, *G. tachinoides*, and *G. austeni*. Human infective *T. b. rhodesiense* trypanosomes constituted 8% (33/422) of all tsetse-derived trypanosome stabilates (Table 3).

### Derived trypanosome stabilates

These are secondary trypanosomes derived from primary trypanosomes after propagation in either culture or the animal host system. The cryobank has 600 derivatives, of which *2*6 are cloned stabilates. The clones include 16 *T. b. rhodesiense*, six *T. b. brucei*, three *T. evansi*, and one *T. vivax*. Derivatives were mainly prepared from *T. brucei* subsp. and *T. congolense*.

### Isolates characterized using molecular and drug sensitivity techniques

 The existing data on the molecular and drug sensitivity patterns of some of the trypanosome stabilates is shown in Tables 4 and 5, respectively. Trypanosomes not characterized by molecular techniques were assigned their species based on their morphology and animal host. These are now undergoing molecular confirmation.

### Potential uses

The data contained in the KETRI cryobank, including (1) history of isolates, (2) diversity of localities and of sample sources, (3) size, (4) published and unpublished information on the stabilates, and (5) availability of *T. b. gambiense* stabilates susceptible to laboratory Swiss white mice, makes it a unique reference research facility on trypanosomiasis. This collection has potential uses in the development and validation of drugs, vaccines, diagnostics, and interrogation of biological phenomenon such as treatment failures. Studies on the effect of storage on the characteristics of the trypanosomes collected over time has been initiated.

Scientists wishing to collaborate and/or enter into partnership on the use of the biospecimens at the KETRI biobank should contact the Centre directly or through the WIPO Re:Search website (www.wipo.int/research/en/partnership/) for details. This data is published in anticipation that it will attract potential partners and collaborators to invest in this facility and make it self-sustaining. It is anticipated that other institutions working in trypanosomiasis-endemic areas will be encouraged to isolate and cryopreserve parasites during regular surveillance and control of African trypanosomiasis for future research and to avoid loss of vital biological information.

**Box 1. Advantages and Improvements of the Cryobank**

Advantages

- A large well-preserved stock of over 2,000 trypanosome stabilates of economic importance
- A unique collection of viable species of trypanosomes collected from different hosts and countries over a period of more than 50 years
- A collection of clones developed from different species of trypanosomes and availability of trypanosome isolates of mixed infections

Improvements

- Establishment of new networks and/or strengthen the current collaborations for sustained collection and cryopreservation of human and animal infective trypanosome isolates
- Replacement of the current equipment in order to reduce the liquid nitrogen usage and associated costs
- Review guidelines for access to isolates
